# Sexual Function in Women Diagnosed with Hereditary Breast and Ovarian Cancer Syndrome

**DOI:** 10.3390/cancers16142601

**Published:** 2024-07-21

**Authors:** Federico Ferrari, Juri Amonti, Andrea Giannini, Hooman Soleymani Majd, Valentina Zizioli, Giancarlo Tisi, Luigi Della Corte, Emma Bonetti, Elisa Gozzini, Franco Odicino

**Affiliations:** 1 Department of Clinical and Experimental Sciences, University of Brescia, 25136 Brescia, Italy; 2 Unit of Gynecology, Sant’Andrea Hospital, Department of Surgical and Medical Sciences and Translational Medicine, Sapienza University of Rome, 00189 Rome, Italy; 3 Department of Gynecological Oncology, Churchill Hospital, Oxford University Hospitals, Oxford OX3 7LE, UK; 4 Struttura Complessa Ostetricia e Ginecologia, Dipartimento Area della Donna e Materno Infantile, Azienda Socio Sanitaria Territoriale Spedali Civili di Brescia, 25136 Brescia, Italye.gozzini92@gmail.com (E.G.); 5 Department of Neuroscience, Reproductive Sciences and Dentistry, School of Medicine, University of Naples Federico II, 80131 Naples, Italy; luigi.dellacorte@unina.it

**Keywords:** sexual health, sexual dysfunction, hereditary breast and ovarian cancer syndrome, BRCA1, BRCA2, SFQ28

## Abstract

**Simple Summary:**

The sexual well-being of women with hereditary breast and ovarian cancer (HBOC) syndrome is often overlooked by clinicians. The aim of the study is to analyze the correlation between HBOC syndrome itself and the potential risk of sexual dysfunction. Understanding this link could help improve clinical practices and the management and counseling of women, emphasizing the importance of openly addressing sexuality-related issues, providing a more targeted support to women affected by this syndrome.

**Abstract:**

Background: Hereditary breast and ovarian cancer syndrome (HBOC) predisposes women to an increased risk mainly of breast and tubo-ovarian cancer. The aim of the study is to investigate whether being diagnosed with HBOC syndrome is itself a risk factor for sexual dysfunction. Methods: An ad hoc questionnaire, including baseline demographic and clinical data, and the Sexual Function Questionnaire 28 (SFQ28) were administered to HBOC female carriers (study group) and to a control group. Results: After propensity score matching (1:1), we enrolled 202 women, 101 in the study group and 101 in the control group. In a multivariate analysis, we finally found that menopausal status was the only risk factor for a significant low score in the domains Desire (HR 0.66; CI95% 0.47–0.93; *p* = 0.017), Arousal (Lubrication) (HR 0.52; CI95% 0.34–0.80; *p* = 0.003), Arousal (Cognitive) (HR 0.64; CI95% 0.44–0.95; *p* = 0.027), and Orgasm (HR 0.33; CI95% (0.16–0.70; *p* = 0.004), independent of risk-reducing surgery for gynecological malignancy. Psycho-oncology support is a protective factor for the Enjoyment domain (HR 1.38; CI95% 1.05–1.81; *p* = 0.022). Conclusions: HBOC syndrome itself does not affect SFQ28 domains, while menopausal status significantly influences sexual health, with potential mitigating effects of psycho-oncological support.

## 1. Introduction

The quality of life in women affected by hereditary breast and ovarian cancer (HBOC) syndrome is not rarely compromised [1,2]. This syndrome is characterized by an elevated risk of developing breast and tubo-ovarian cancer, frequently associated with mutations in various genes, of which BRCA1 and BRCA2 are the most frequent [3]. These patients not only face an increased risk of these malignancies, but also experience their onset at a younger age compared to the general population [4].

Risk-reducing breast and gynecological surgeries (respectively bilateral mastectomy and bilateral salpingo-oophorectomy) have become established preventive approaches to mitigate oncological risks, albeit not infrequently associated with a decline in women’s sexual quality of life [1,5]. This aspect is crucial for overall quality of life, but it is often overlooked by the clinicians, in particular during the first counseling for a diagnosis of HBOC syndrome regarding risk-reducing approaches and their subsequent management of the consequences [1,6]. Sexual dysfunction is a term that encompasses various issues regarding the sexual activity and satisfaction of the women, impacting their quality of life, emotional well-being, and relationships. The details of a potential sexual dysfunction can be objectively studied using a validated questionnaire, the Sexual Female Health Questionnaire 28 (SFQ28), that can investigate multiple domains regarding sexual health, such as Desire, Arousal (Sensation), Arousal (Lubrication), Orgasm, Pain, Enjoyment, and the Role of the Partner [7].

Chan et al. highlighted that BRCA-mutated women who underwent risk-reducing oophorectomy were more likely to experience sexual dysfunction [5]. At our institution, the counseling of these women takes into deep account the side effect of the eventual iatrogenic menopause and the psychological aspects related to bilateral mastectomy and the strategies to overcome and manage the side effects. The sexual health after risk-reducing surgery (RSS) is a point of concern for the women when they are informed about the surgical preventive strategies, but often, this topic is not deeply explored during counseling when compared to oncological risk and contraceptive and fertility issues [8,9,10,11]. We noted (unpublished data) in running a dedicated outpatient clinic for HBOC syndrome carriers frequent negative spontaneous feedback regarding a problem with a normal sexual function, also in those who did not undergo RRS for gynecological cancer.

The aim of this study is to investigate whether being diagnosed with an HBOC syndrome can itself adversely impact sexual health and the role of a diagnosis of breast or tubo-ovarian cancer.

## 2. Materials and Methods

We conducted a cross-sectional survey in a tertiary academic hospital (University of Brescia, Brescia), enrolling women, aged at least 18 years old, who were referred to our dedicated outpatient clinic for HBCOC syndrome, from January 2022 to January 2023 (study group). In the same period, we enrolled a control group of women working at our hospital institution and the female staff of the local University of Brescia.

At our institution, the outpatient clinic for HBOC syndrome is a multidisciplinary evaluation offered to all women with a confirmed diagnosis of a germline mutation that are referred mainly from the departments of surgical oncology, medical oncology, and gynecology; from other hospitals; from general medical practitioners; and also self-presented. During this evaluation, a medical oncologist, a breast surgeon, a geneticist, a psycho-oncologist, and an oncological gynecologist discuss with the woman about the significance of being a carrier of HBOC syndrome and the next steps in terms of preventative options and surveillance. The psycho-oncologist support is offered to all women.

All the data used in this manuscript derive from an anonymous questionnaire and hence are already anonymized at the moment of data collection, and the local ethical committee of Brescia considered the study exempt. The design, analysis, interpretation of data, drafting, and revisions conform to the Helsinki Declaration, the Committee on Publication Ethics guidelines (http://publicationethics.org/, accessed on 19 July 2024), and the REporting of studies Conducted using Observational Routinely collected health Data (RECORD) statement, validated by the Enhancing the Quality and Transparency of Health Research Network (www.equator-network.org, accessed on 19 July 2024). No personal data that could lead to the formal identification of the patient were stored in the databases. The study was not advertised.

We administered a dedicated ad hoc questionnaire investigating baseline characteristics and the Sexual Female Health Questionnaire 28 (SFQ28) (Supplementary Materials). We collected baseline demographic (age, education level, occupation, nulligravida rates, and presence of a stable partner) and clinical (such as the type of HBOC-related mutation, the actual medication for a psychiatric condition including depression, menopausal status, the eventual use of hormonal replacement treatment—HRT, the eventual risk-reducing procedure, eventual diagnosis of breast or tubo-ovarian cancer, and the access to a psycho-oncological support) characteristics. In detail, the menopausal status was defined at least 12 months after RRS for gynecological cancer or at least 12 months from the last period.

We performed propensity score matching with a 1:1 ratio between matched subjects (namely those enrolled in the study group and the control group), using nearest neighbor matching with a caliper width of a 0.20 standardized mean difference of the logit of the propensity scores using age and the presence of a perceived stable partner.

The SFQ28 questionnaire serves as a comprehensive evaluation tool for female sexual function, which is divided into eight domains (Desire, Arousal (Sensation), Arousal (Lubrication), Orgasm, Pain, Enjoyment, and Partner) [12]. While it lacks a composite score, specific cut-off scores for each domain have been established, showing the likelihood of dysfunction—ranging from low probability to high probability. A higher score on the SFQ28 reflects a better sexual function. This questionnaire has been translated into Italian [10,13]. We administered the questionnaire at least six months after the eventual diagnosis of HBOC syndrome and at least six months after the treatment for gynecological or breast cancer.

We used independent sample *t*-tests to compare continuous variables or the Mann–Whitney U-test as appropriate, based on data distribution. We used the Pearson Chi-Square or Fisher’s extract test for categorical variables. Multivariate analysis was performed using a general linear model and data were normalized as appropriate. All statistical analyses were performed using IBM © SPSS Statistics 22.0. Statistical significance was considered for *p* < 0.05.

## 3. Results

During the survey period, we administered the questionnaire to 423 women with a response rate of 63.3% (268 women). After propensity score matching, we finally enrolled 202 women (Table 1). 

No differences were noted in age, nulligravida rates, menopausal status, the use of HRT, the education level, an ongoing medication for a psychiatric condition, and the presence of a perceived stable partner. No history of alcohol abuse was registered. We found instead a significant difference in the diagnosis rates of breast and/or tubo-ovarian cancer, that was obviously higher in the study group (50.5% versus 4.95%; *p* < 0.01). In this group, the rates of RRS for breast cancer are lower compared to RRS for tubo-ovarian cancer (15% versus 40%, respectively) and active psycho-oncology support was more frequent in the study group. Overall, in the study group, 30 women with menopausal status (48.4%) underwent surgery that bilaterally removed the adnexa (either for RRS or for surgical treatment).

Using the validated SFQ28 questionnaire, the collected data on the eight domains (Desire, Arousal (Sensation), Arousal (Lubrication), Arousal (Cognitive), Orgasm, Pain, Enjoyment, and Partner) were compared between the two groups.

The domains that were found to be significantly different between the two groups of patients are Desire (*p* < 0.01), Arousal (Lubrication) (*p* < 0.01), Arousal (Cognitive) (*p* < 0.0), Orgasm (*p* = 0.02), pain (*p* = 0.01), and Enjoyment (*p* < 0.01) as reported in Table 2.

We selected the domains in which we found a significant difference according to Table 1 and undertook a multivariate analysis considering the mutational status, menopause, education level, and the diagnosis of cancer.

In the multivariate analysis, we observed that being diagnosed with HBOC syndrome does not significantly decrease the score in the Desire, Arousal (Lubrication), Arousal (Cognitive), Orgasm, Pain, and Enjoyment domains (Table 2). Other factors that entail a risk of a decrease in the considered domains are the menopausal status and the diagnosis of cancer. In particular, the menopausal status plays a predominant role as an adverse factor, resulting in an increased risk for low scores, most clear for the Desire domain (HR 0.03; CI95% 0.01–0.11; *p* < 0.01). Also, the diagnosis of cancer, regardless of the mutational status, independently impacts the risk for a low score for the Arousal (Cognitive), Orgasm, and Enjoyment domains (Table 3).

In Table 4, we report the score of each domain for the women diagnosed with an HBOC syndrome (namely the study group), categorized according to the diagnosis of cancer.

Based on Table 4, a further exploratory multivariate analysis was conducted in the study group, taking into account age, menopausal status, the history of RRS either for breast and/or tubo-ovarian cancer, and the psycho-oncology support for each domain that scored significantly different among those women with and without a diagnosis of cancer, namely, Desire, Arousal (Lubrication), Arousal (Cognitive), Orgasm, and Enjoyment (Table 5).

Based on the multivariate analysis, in women with HBCO syndrome, menopause has been the only risk factor for a low score in most of the aforementioned domains, except for the Enjoyment domain. Interestingly, in the Enjoyment domain, psycho-oncology support is the sole independent factor that increases the chance of a higher score (HR 1.38; CI95% 1.05–1.81; *p* = 0.022). Age seems to play a role as a risk factor for the domain Arousal (Lubrication) with borderline statistical significance.

## 4. Discussion

Women diagnosed with HBOC syndrome are not exposed to a greater risk of sexual health dysfunction. Menopausal status increases the risk of low scores in most of the domains of the SFQ28 questionnaire as a predominantly independent factor. The diagnosis of cancer among women affected by HBOC syndrome does not impact the scores of the questionnaire, resulting in a non-significant risk factor for sexual health dysfunction. These findings are partially confirmed by the literature [14].

In our study, menopausal status particularly determines a risk rate of dysfunction in the Desire, Arousal (Lubrication), Arousal (Cognitive), and Orgasm domains in women with HBOC syndrome. This phenomenon can be explained, as shown in previous studies, because the decline in estrogen levels induces typical symptoms, including reduced lubrication and consequent increased pain during intercourse [7,15,16], regardless of the mutational status. In fact, after premature ovarian failure, nearly 50% of the women refer a low sexual desire, the majority reports vaginal dryness, 17–42% experience pain or discomfort during sex, and nearly 60% cannot reach an orgasm. To relieve these genitourinary symptoms, lubricants and moisturizers are often prescribed, whereas the most efficient strategy is a topical treatment with estrogens [17,18].

However, women with HBOC syndrome, on average, experience menopause earlier than usual, and based on genome-wide association studies, it is known that being a carrier of BRCA1 or BRCA2 gene mutation is related with increased ovarian aging [6,19]. The management of early or iatrogenic menopause is challenging and a multidisciplinary team should carefully inform the woman about the opportunity for hormonal and non-estrogenic therapies and should take in account the adoption of RSS strategies, either for breast or gynecological malignancies and the eventual diagnosis of cancer [1,20,21,22].

At the population level, the diagnosis of cancer negatively influences the Arousal (Cognitive), Orgasm, and Enjoyment domains, with an increased risk rate of experiencing sexual dysfunction, likely linked to the development of anxiety, depression, or high levels of stress in women receiving a cancer diagnosis, which are associated with an increased risk of female sexual dysfunction. Similar findings were confirmed by Watts et al., demonstrating that the prevalence of depression and anxiety in women with ovarian cancer, across the treatment spectrum, is significantly greater than in the healthy female population [23]. Oncological treatments, both medical and surgical, to which patients are subjected, result in physical modifications and/or side effects that can negatively impact certain aspects of sexuality, such as the use of radiotherapy and the adoption of aggressive surgery, resulting also in potential physical damage when conservative surgery is not workable [4,24,25,26]. However, based on our multivariate analyses, we finally found that the main risk factor in women diagnosed with HBOC syndrome is still the menopausal status, which can result from the iatrogenic effect of RRS or directly from cancer treatment. In fact, in our study, the role of RRS for gynecological malignancy failed to show an increased risk for sexual dysfunction and this finding is in contrast with the literature, where this type of surgery is suggested to decrease sexual health [27]. This result can be partially explained by the role of the psycho-oncology support, which significantly impacts, as a protective factor, the Enjoyment domain (HR 1.38; CI95% 1.05–1.81; *p* = 0.022). Even though the rate of HRT users is very low at the population level and was not included in the multivariate analysis, it can, however, contribute to decreased sexual dysfunction, especially in those women experiencing early or iatrogenic menopause. In fact, two systematic reviews suggested that the use of HRT in women with HBOC syndrome does not impact the risk of breast cancer and that its use can be held until the natural onset of menopause [28].

The results of our study are partially in contrast with a review by Kershaw et al. that reported how the sexual function declines after RRS for gynecological malignancy, independent of the menopausal status, even though no mention regarding the use of HRT was taken in account [9]. On the contrary, Gasparri et al. support that an anticipated lack of sexual hormones impacts various systems with a detrimental effect and that HRT is the only effective strategy to overcome the consequences of a menopausal status [29].

The strengths of our study are based on the enrollment of a healthy control group, that was corrected by a propensity score match with the administration of a dedicated questionnaire for sexual function investigating the presence of a stable partner, level of education, and occupational status as a surrogate for low-income information and the presence of a psychiatric condition, including depression. The limitations of the study are the relatively low sample size and the lack of a prospective follow-up, to understand the trend of the scores of SFQ28 over time. Moreover, we cannot say anything regarding the perimenopausal status of the women, since these patients were excluded from our initial criteria. Again, dissatisfaction with sleep and a worse perception of global health are well-known predictive factors for sexual dysfunction in climacteric women but unfortunately they were unexplored in our study [30]. However, we say that since we enrolled a group of women affected by HBCO syndrome and eventually treated for breast and/or tubo-ovarian cancer, investigating the perception of a worse global health could have largely impacted well beyond objective risk factors, and hence we avoided this question on purpose. Again, any further data regarding the presence of a double germline BRCA mutation are not clinically relevant given the low rate of this particular condition. Lastly, this is a monocentric study, with a Caucasian population that may create an inferential bias in the conclusion, not hence applicable to other ethnicities.

## 5. Conclusions

This study aimed to show if the diagnosis of HBCO syndrome can impact in itself the sexual health of female patients. According to our findings, being diagnosed with an increased risk of developing cancer does not influence any of the eight domains of the SFQ28 questionnaire. However, the quest for timely management of the effects of menopause should be incorporated in the first counseling of a woman diagnosed with HBOC syndrome, since most of the attention is focused on enhanced surveillance, preventive medications, risk-reducing mastectomy and reconstruction, risk-reducing bilateral salpingo-oophorectomy, fertility, and only lastly on sexuality and menopausal management.

## Figures and Tables

**Table 1:** Baseline demographic and clinical characteristics.

	Population (*n* = 202)	Study Group (*n* = 101)	Control Group (*n* = 101)	Standardized Mean Difference	*p*
Age	47 (35–55.5)	47 (35–56)	47 (35–55)	0.16	0.87
BMI	26.8 (21.6–31)	26.4 (21.4–31)	27.1 (22–30.1)	-	0.67
Active smoking	23 (11.4%)	12 (11.8%)	11 (10.8%)	-	0.77
Nulligravida	66 (32.7%)	34 (33.7%)	31 (68.3%)	-	0.38
Menopause	122 (61.3%)	62 (61.3%)	38 (37.6%)	-	<0.01
Menopausal age	49 (47–52)	48 (41–50.3)	50 (49–53)	-	<0.01
HRT	25 (12,4%)	14 (13.9%)	11 (10.9%)	-	0.63
Higher education level *	155 (76.3%)	76 (75.2%)	88 (86.4%)	-	<0.01
Psychiatric condition	7 (3.5%)	3 (2.9%)	4 (3.9%)	-	0.64
Partner	182 (90.1%)	91 (90.1%) *	91 (90.1%)	0.01	0.31
Unoccupied	3 (1.4%)	2 (1.8%)	1 (1%)	-	<0.67
Mutational status	101 (50%)	101 (100%)	-	-	-
BRCA 1	42 (20.8%)	42 (41.6%)			
BRCA 2	44 (21.8%)	44 (43.6%)			
BRCA 1 and BRCA 2	8 (3.9%)	8 (7.9%)			
RAD51c	2 (1%)	2 (2.0%)			
ATM	1 (0.5%)	1 (1.0%)			
Chek2	1 (0.5%)	1 (1.0%)			
PALB2	1 (0.5%)	1 (1.0%)			
Pot1	1 (0.5%)	1 (1.0%)			
Li-Fraumeni (TP53 mutated)	1 (0.5%)	1 (1.0%)			
RRS for Breast Cancer	15 (15.0%)	15 (15.0%)	-	-	-
RRS for Tubo-Ovarian Cancer	40 (40.0%) *	40 (40.0%) *	-	-	-
Diagnosis of cancer	56 (20.9%)	51 (50.5%)	5 (4.95%)	-	<0.01
Breast	44 (16.4%)	40 (39.6%)	4 (3.95%)		
Tubo-ovarian	8 (3.0%)	7 (6.9%)	1 (1%)		
Breast and tubo-ovarian	4 (1.5%)	4 (4.0%)	-		
Active psycho-oncology support	20 (9.9%)	19 (18.8%)	1 (1%)	-	-

Values are presented as median and interquartile range (IQR) or absolute count and rate. BMI: body mass index; HRT = hormone replacement treatment; RRS = risk-reducing surgery. * includes undergraduate and postgraduate education. + consensual total hysterectomy was performed in 7 patients during RRS.

**Table 2:** Score for each domain of the questionnaire in the whole population, study group, and control group.

	Population (*n* = 202)	Study Group (*n* = 101)	Control Group (*n* = 101)	*p*
Desire	15.5 (5.2)	14.5 (5.8)	16.0 (4.7)	<0.01
Arousal (Sensation)	8.3 (5.2)	7.7 (5.2)	8.7 (5.2)	0.18
Arousal (Lubrication)	5.0 (3.0)	4.4 (3.1)	5.4 (2.9)	<0.01
Arousal (Cognitive)	4.8 (2.8)	4.2 (2.7)	5.1 (2.7)	<0.01
Orgasm	8.4 (5.0)	7.3 (5.3)	9.1 (4.7)	0.01
Pain	10.8 (5.4)	9.7 (5.8)	11.5 (5.0)	0.01
Enjoyment	16.4 (7.8)	14.4 (7.9)	17.5 (7.5)	0.01
Partner	8.4 (2.4)	8.4 (2.1)	8.3 (2.6)	0.12

Values are presented as means (DS).

**Table 3:** Multivariate analysis in the whole population.

	Mutational Status	Menopause	High Education Level	Diagnosis of Cancer
Desire	HR 0.93 (CI95% 0.21–4.14) *p* = 0.925	HR 0.03 (CI95% 0.01–0.11) *p* = 0.0001	HR 1.18 (CI95% 0.20–6.79) *p* = 0.854	HR 0.25 (Cl95% 0.03–1.82) *p* = 0.169
Arousal (Lubrication)	HR 0.98 (CI95% 0.84–1.14) *p* = 0.781	HR 0.62 (CI95% 0.54–0.71) *p* = 0.0001	HR 1.03 (CI95% 0.86–1.24) *p* = 0.711	HR 0.83 (CI95% 0.67–1.01) *p* = 0.069
Arousal (Cognitive)	HR 0.95 (CI95% 0.83–1.08) *p* = 0.408	HR 0.74 (CI95% 0.66–0.83) *p* = 0.0001	HR 1.07 (CI95% 0.92–1.25) *p* = 0.380	HR 0.82 (CI95% 0.69–0.98) *p* = 0.026
Orgasm	HR 1.00 (CI95% 0.81–1.23) *p* = 0.964	HR 0.78 (CI95% 0.65–0.95) *p* = 0.011	HR 0.94 (CI95% 0.74–1.21) *p* = 0.644	HR 0.70 (CI95% 0.52–0.93) *p* = 0.015
Pain	HR 0.95 (CI95% 0.87–1.04) *p* = 0.298	HR 0.91 (Cl95% 0.84–0.99) *p* = 0.026	HR 1.00 (CI95% 0.90–112) *p* = 0.974	HR 0.94 (CI95% 0.83–1.06) *p* = 0.298
Enjoyment	HR 0.90 (CI95% 0.69–1.15) *p* = 0.393	HR 0.71 (CI95% 0.56–0.89) *p* = 0.003	HR 1.01 (CI95% 0.75–1.36) *p* = 0.945	HR 0.71 (CI95% 0.51–1.00) *p* = 0.049

HR: hazard ratio; CI95%: confidence interval at 95%.

**Table 4:** Score for each domain of the questionnaire in the study group.

	Diagnosis of Cancer (*n* = 51)	No Cancer Diagnosis (*n* = 50)	*p*
Desire	12.9 (5.4)	16.22 (5.6)	0.03
Arousal (Sensation)	7.20 (5.5)	8.28 (4.8)	0.29
Arousal (Lubrication)	3.57 (2.9)	5.26 (3.18)	<0.01
Arousal (Cognitive)	3.55 (2.6)	4.86 (2.6)	0.02
Orgasm	6.04 (5.2)	8.61 (5.2)	0.02
Pain	8.73 (5.8)	10.74 (5.6)	0.08
Enjoyment	12.69 (8)	16.24 (7.3)	0.02
Partner	8.69 (1.9)	8.12 (2.3)	0.18

Values are presented as means (DS).

**Table 5:** Multivariate analysis in the study group.

	Diagnosis of Cancer	Age	Menopausal Status	RSS Breast	RSS Gynecological	Active Psycho-Oncology Support
Desire	HR 1.13 (Cl95% 0.92–1.40) *p* = 0.239	HR 0.99 (CI95% 0.98–1.00) *p* = 0.163	HR 0.66 (Cl95% 0.47–0.93) *p* = 0.017	HR 1.12 (CI95% 0.88–1.44) *p* = 0.355	HR 1.11 (CI95% 0.90–1.36) *p* = 0.330	HR 0.84 (Cl95% 0.67–1.05) *p* = 0.129
Arousal (Lubrication)	HR 1.15 (CI95% 0.87–1.54) *p* = 0.318	HR 0.99 (CI95% 0.97–1.00) *p* = 0.05	HR 0.52 (CI95% 0.34–0.80) *p* = 0.003	HR 0.97 (CI95% 0.70–1.36) *p* = 0.872	HR 1.04 (CI95% 0.80–1.36) *p* = 0.741	HR 0.88 (CI95% 0.65–1.19) *p* = 0.391
Arousal (Cognitive)	HR 1.01 (CI95% 0.79–1.31) *p* = 0.913	HR 0.99 (CI95% 0.98–1.01) *p* = 0.290	HR 0.64 (CI95% 0.44–0.95) *p* = 0.027	HR 1.01 (CI95% 0.75–1.36) *p* = 0.933	HR 1.13 (CI95% 0.89–1.44) *p* = 0.320	HR 0.95 (CI95% 0.72–1.24) *p* = 0.693
Orgasm	HR 0.87 (CI95% 0.52–1.46) *p* = 0.599	HR 1.02 (CI95% 0.99–1.04) *p* = 0.167	HR 0.33 (CI95% 0.16–0.70) *p* = 0.004	HR 0.97 (CI95% (0.55–1.73) *p* = 0.927	HR 1.57 (CI95% 0.96–2.56) *p* = 0.069	HR 1.15 (CI95% 0.67–1.96) *p* = 0.609
Enjoyment	HR 0.98 (CI95% 0.61–1.57) *p* = 0.927	HR 0.99 (CI95% 0.97–1.02) *p* = 0.589	HR 0.70 (CI95% 0.33–1.50) *p* = 0.359	HR 1.04 (CI95% 0.61–1.80) *p* = 0.876	HR 0.92 (CI95% 0.57–1.46) *p* = 0.712	HR 1.38 (CI95% 1.05–1.81) *p* = 0.022

HR: hazard ratio; CI95%: confidence interval at 95%.

## Data Availability

The data presented in this study are available on request from the corresponding author. The data are not publicly available due to privacy or ethical restrictions.

## Supplementary Materials

The following supporting information can be downloaded at: https://www.mdpi.com/article/10.3390/cancers16142601/s1.
