# Supplementary material for: Sexual Function in Women Diagnosed with Hereditary Breast and Ovarian Cancer Syndrome
Source: Cancers (Basel). 2024 Jul 21;16(14):2601. doi: 10.3390/cancers16142601 (PMC11274699; doi:10.3390/cancers16142601)
Supplement: Supplementary file 1 [file cancers-16-02601-s001.zip › cancers-3064655-supplementary.pdf]

Baseline characteristics

What is your employment status?

- Housewife
- Worker
- Employee
- Self-employed
- Manager
- Unemployed
- Student

What is your education level?

- Elementary school diploma
- Middle school diploma
- High school diploma
- University degree
- None

What is your current age? __

Are you in menopause?

- Yes
- No

If yes, at what age did you enter menopause? __

If you are in menopause, have you ever taken hormone replacement therapy (HRT)?

- Yes
- No
- I am not in menopause

Have you had one or more children?

- Yes
- No

If you have had children, do they still live with you?

- Yes
- No
- I have not had children

Do you have a stable partner (feeling “stable relationship”)?

- Yes
- No

Do you have a mutation in the following genes:

- BRCA 1
- BRCA 2
- I do not have a mutation
- Other genes:

Enter the (approximate) date when you were informed of being a mutation carrier: ___

Were you offered psychological support?

- Yes, but I did not use it
- Yes, I used it
- No

Have you undergone risk-reducing surgery?

- Yes, for gynecological cancer risk
- Yes, for breast cancer risk
- No

If yes, on what date did you undergo risk-reducing surgery for gynecological cancer? ___

If yes, on what date did you undergo risk-reducing surgery for breast cancer? ___

If you have undergone mastectomy/quadrantectomy, have you had breast reconstruction surgery (e.g., insertion of implants/expanders, lipofilling...)?

- Yes
- No
- I have not undergone mastectomy/quadrantectomy

Have you received a diagnosis of cancer:

- Breast
- Ovarian and/or tubal
- None

If yes, on what date did you receive the diagnosis of breast cancer? ___

If yes, on what date did you receive the diagnosis of ovarian and/or tubal cancer? ___

Are you currently taking medication or undergoing treatment for ovarian and/or breast cancer?

- Yes
- No

Do you suffer from any psychiatric conditions (including depression on medication)?

- Yes
- No

Have you ever experienced trauma or sexual abuse?

- Yes
- No
